# Supplementary material for: Design of a water-soluble transmembrane receptor kinase with intact molecular function by QTY code
Source: Nat Commun. 2024 Jun 10;15:4293. doi: 10.1038/s41467-024-48513-9 (PMC11164701; doi:10.1038/s41467-024-48513-9)
Supplement: Supplementary file 1 — Supplementary Information [file 41467_2024_48513_MOESM1_ESM.pdf]

Supplementary Information

Design of a water-solubilized transmembrane receptor kinase with intact molecular function by QTY code

Mengke Li[1,2], Hongzhi Tang[1], Rui Qing[1], Yanze Wang[3], Jiongqin Liu[1], Rui Wang[1], Shan Lyu[1], Lina Ma[1], Ping Xu[1]*, Shuguang Zhang[2]*, Fei Tao[1]*

[1]State Key Laboratory of Microbial Metabolism, Joint International Research Laboratory of Metabolic and Developmental Sciences, School of Life Sciences and Biotechnology, Shanghai Jiao Tong University, Shanghai 200240, China

[2]Laboratory of Molecular Architecture, Media Lab, Massachusetts Institute of Technology, Cambridge, MA 02139, USA

[3]Department of Chemistry, Massachusetts Institute of Technology, Cambridge, MA 02139, USA

*Correspondence: taofei@sjtu.edu.cn (F.T.), shuguang@mit.edu (S.Z.), pingxu@sjtu.edu.cn (P.X.)

a

Protein	pI	MW (kDa)	Variation rate (%)	Variation rate (TM, %)
CpxA	5.61	51.6	-	-
$CpxA^{QTY}$	5.61	51.9	5.47%	58.1%

b

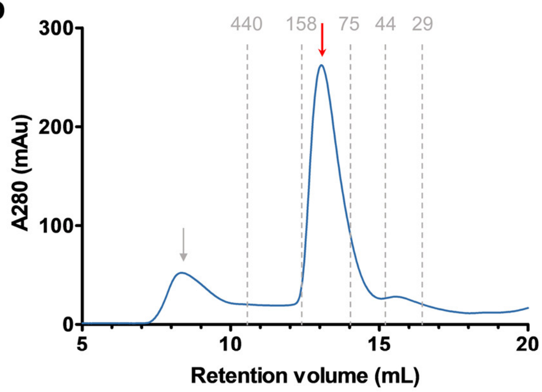

c

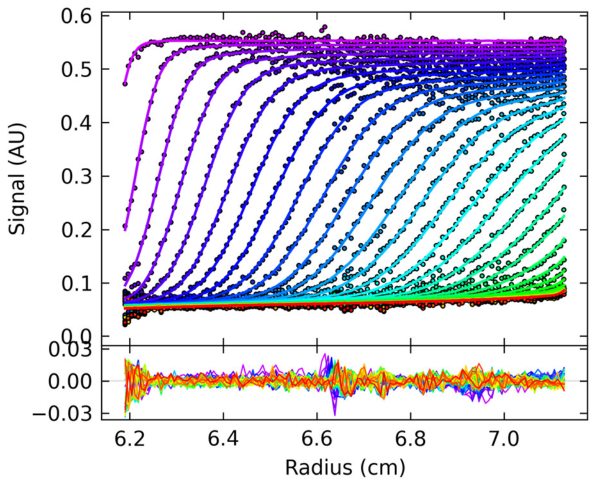

d

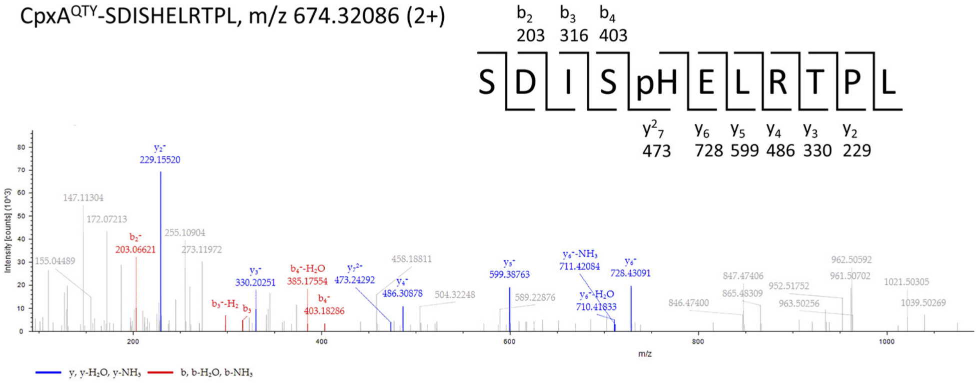

e

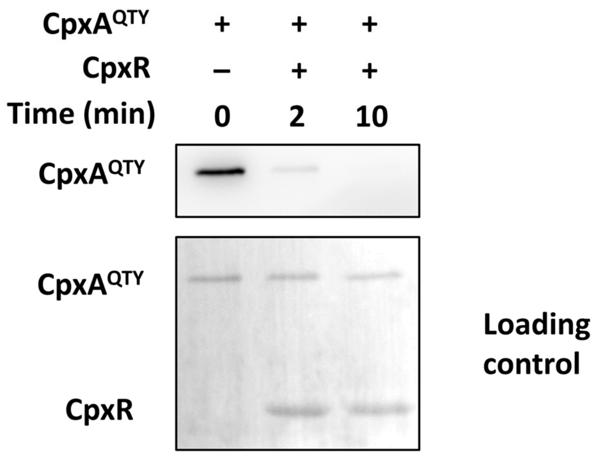

Supplementary Figure 1 | Extended Data of the characterizations of CpxA^{QTY}. a, Characteristics of CpxA and CpxA^{QTY} with isoelectric point (pI), molecular weight (MW), total variation rate, and transmembrane (TM) variation rate. **b**, Size exclusion chromatography results of CpxA^{QTY}. Grey dashed lines denote the retention volumes of the standard proteins, labelled with the corresponding molecular weight (kDa). Red arrows denote the dimers, grey arrows denote aggregates. The identities of the peak fractions were identified by SDS-PAGE. Superdex 200 (Cytiva Life Sciences) was used for SEC. **c**, Sedimentation velocity AUC data and fitted single-ideal species model curves (top) and residuals for the above fit (bottom). Every fitted curve in a color stands for a group of scanning data and the corresponding residuals were calculated. The figure was made by the software GUSSI. **d**, Mass spectrometry results of CpxA^{QTY} identified the phosphorylation of expected phosphorylation site. **e**, Phosphotransferase activity of CpxA^{QTY} characterized by anti-pHis western blotting. After the autophosphorylation of 1 μM CpxA^{QTY} with 1 mM ATP for 30 min, 4 μM CpxR was added to initiate the phosphotransfer. Source data are provided as a Source Data file.

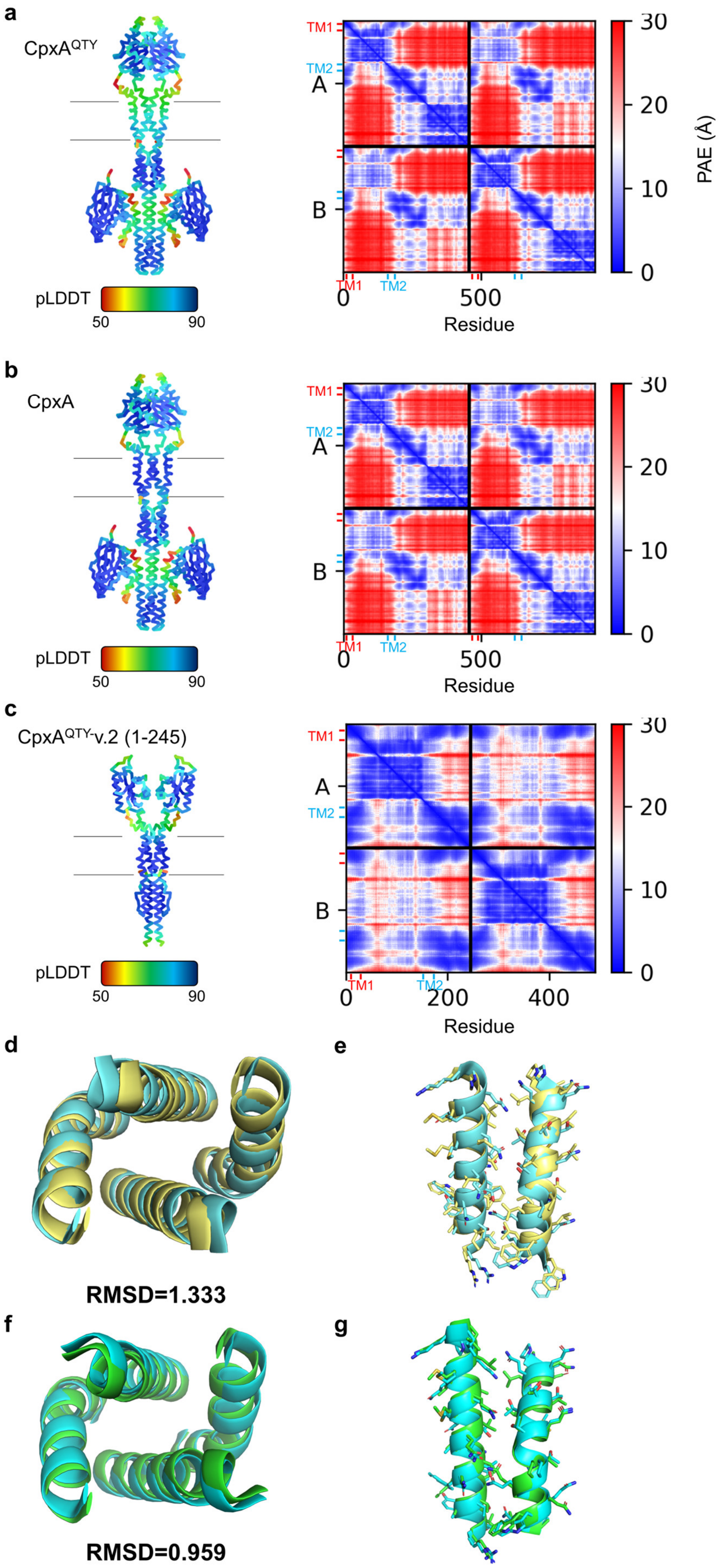
a
CpxA^{QTY}
pLDDT
50
90
TM1
TM2
A
B
0
500
Residue
30
20
10
0
PAE (Å)
b
CpxA
c
CpxA^{QTY}-v.2 (1-245)
200
400
d
RMSD=1.333
e
f
RMSD=0.959
g

Supplementary Figure 2 | AlphaFold2 models. a-c, Prediction quality of the AlphaFold2 structure model of CpxA^{QTY} (**a**), CpxA (**b**) and CpxA^{QTY}-v.2 (**c**, residue 1-245)**.** (Left) pLDDT scores for assessing local structure confidence. The two lines represent the plasma membrane. The pLDDT scale was shown at the bottom. (Right) PAE plots for assessing complex structure confidence. "A" and "B" denotes chain A and B. TM1 (red) and TM2 (cyan) has been labeled. **d**, Superposition of the transmembrane four-helix bundle of AlphaFold2 models of CpxA^{QTY} (cyan) and CpxA (yellow), as viewed from the periplasm looking into the cytoplasm. **e**, Side view of superposition of the monomeric transmembrane domain of AlphaFold2 models of CpxA^{QTY} and CpxA, with side chains shown. **f-g**, Superposition of the transmembrane four-helix bundle of AlphaFold2 models of CpxA^{QTY} (cyan) and CpxA^{QTY}-v.2 (green). The display style is the same as in **d-e**.

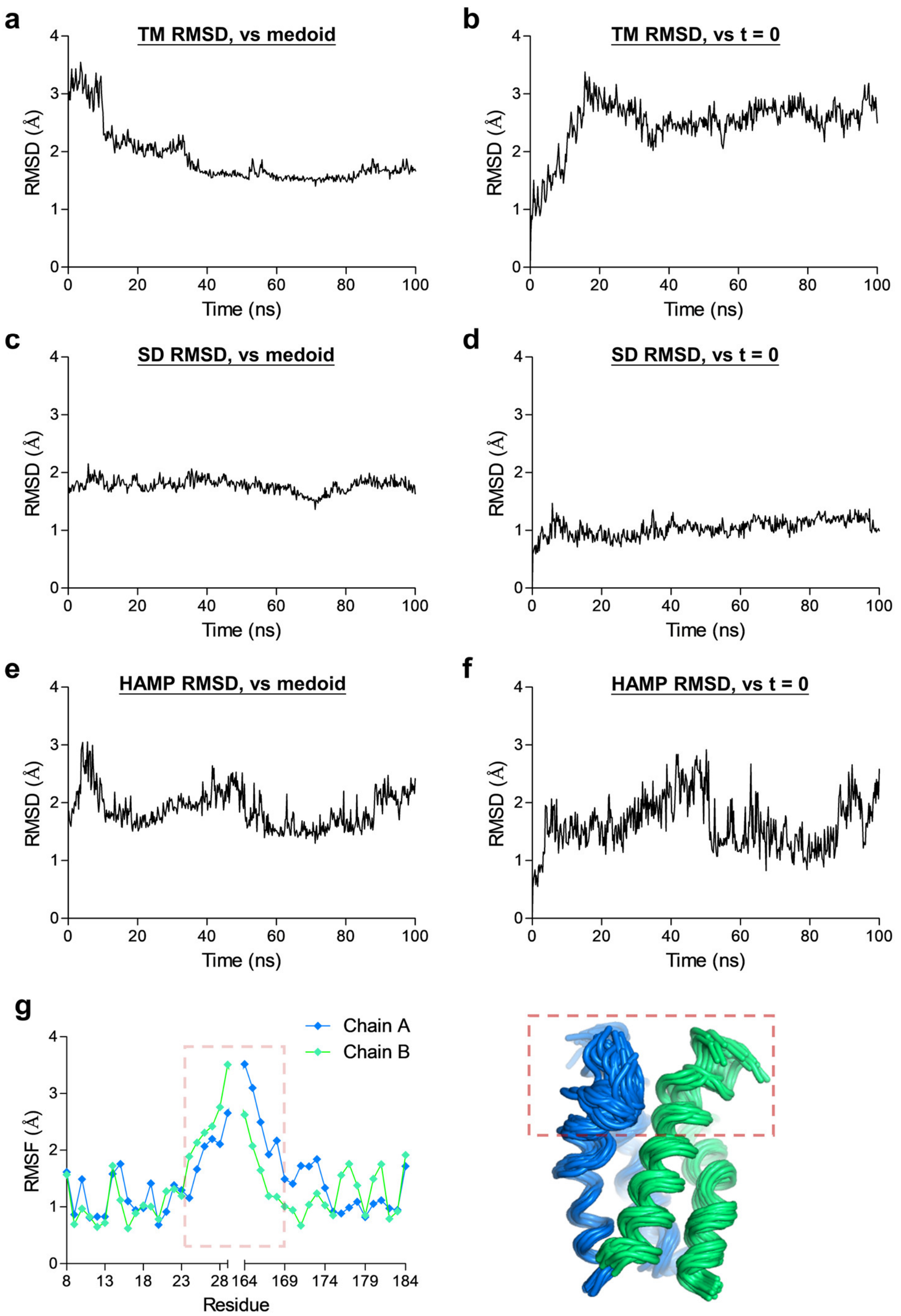

Supplementary Figure 3 | RMSD and RMSF curves in MD simulations of CpxA^{QTY}. Backbone RMSD of the transmembrane domain, versus medoid frame (**a**) or versus starting frame (**b**); Backbone RMSD of the sensor domain, versus medoid frame (**c**) or versus starting frame (**d**); Backbone RMSD of the HAMP domain, versus medoid frame (**e**) or versus starting frame (**f**). **g**, (Left) RMSF of the transmembrane domain, versus medoid frame. (Right) The structures of the transmembrane domain extracted every 2 ns are aligned, highlighting the flexibility of the periplasm-facing region (indicated by the red dashed rectangle). Chain A is in blue, and B in green. Source data are provided as a Source Data file.

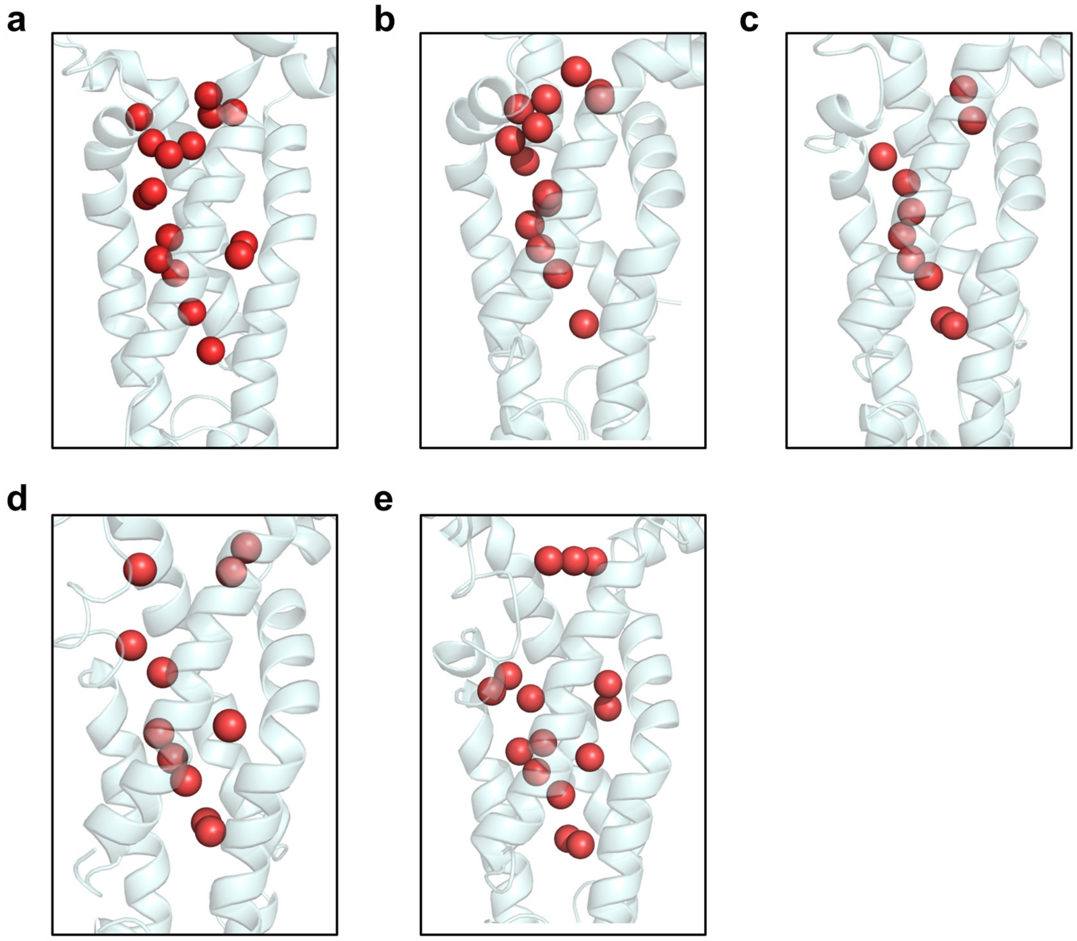

Supplementary Figure 4 | Profiles of water molecule distribution inside the transmembrane domain in MD simulation of CpxA^{QTY}. a-e, Snapshots of 10 ns, 30 ns, 50 ns, 70 ns and 90 ns, respectively.

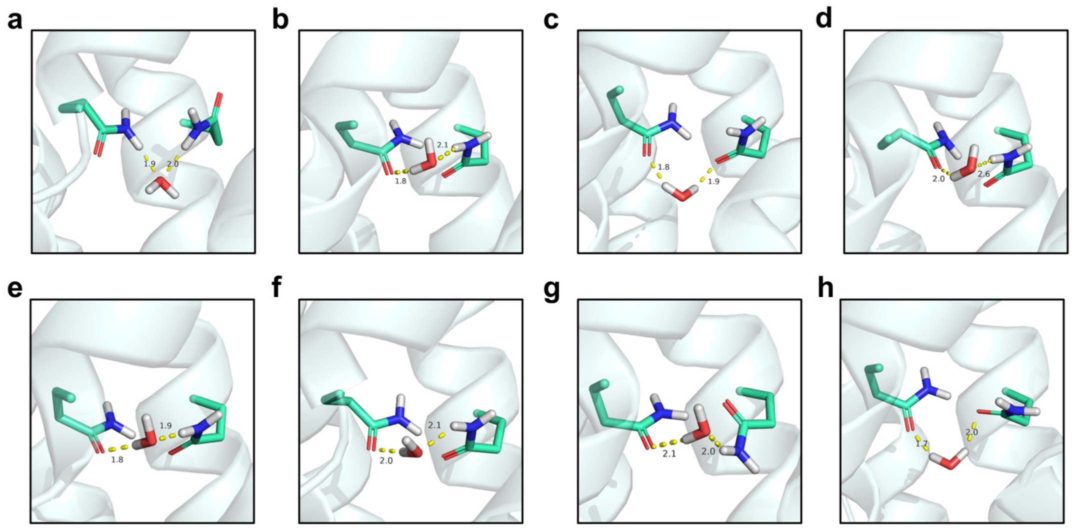

Supplementary Figure 5 | A water molecule formed H-bonds with Q17 of both chains for most time of the simulation. a-h, The water-mediated interaction between Q17 in both chains in different simulation frames. Snapshots of 30 ns, 40 ns, 50 ns, 60 ns, 70 ns, 82 ns, 90 ns and 100 ns, respectively.

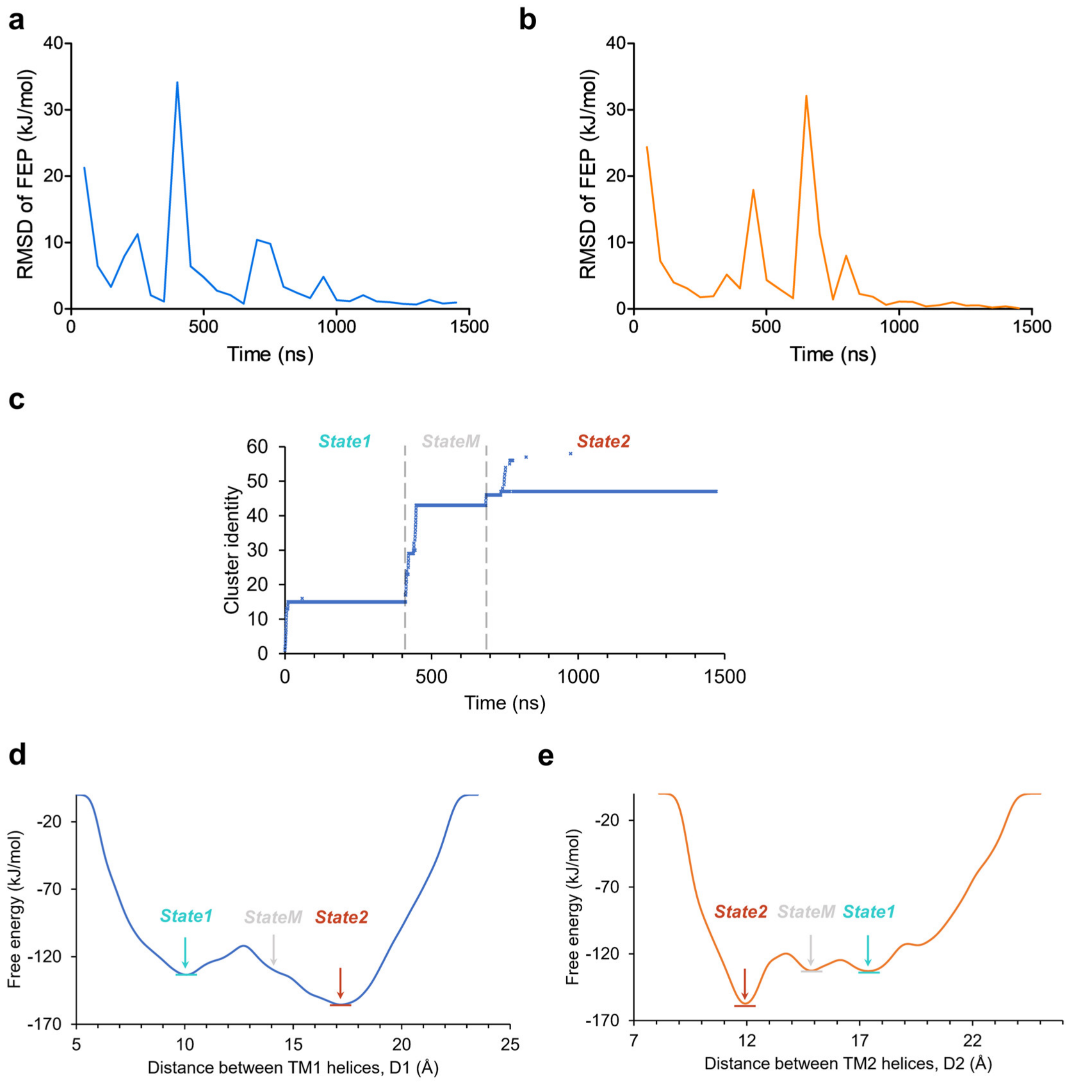

Supplementary Figure 6 | Some extended data of the PBMetaD simulation of CpxA^{QTY}. **a-b**, Convergence of the PBMetaD simulation was shown by RMSD of the FEP of D1 (**a**) and D2 (**b**). The FEP was aligned every 50 ns. **c,** The clustering of the conformations across the simulation time identified three major clusters, corresponding to the three states shown in CV-time curves, Fig. 6b. **d-e**, Free energy profile (FEP) of D1 (**d**) and D2 (**e**). The free energy minima were indicated by arrows and classified into three states. Source data are provided as a Source Data file.

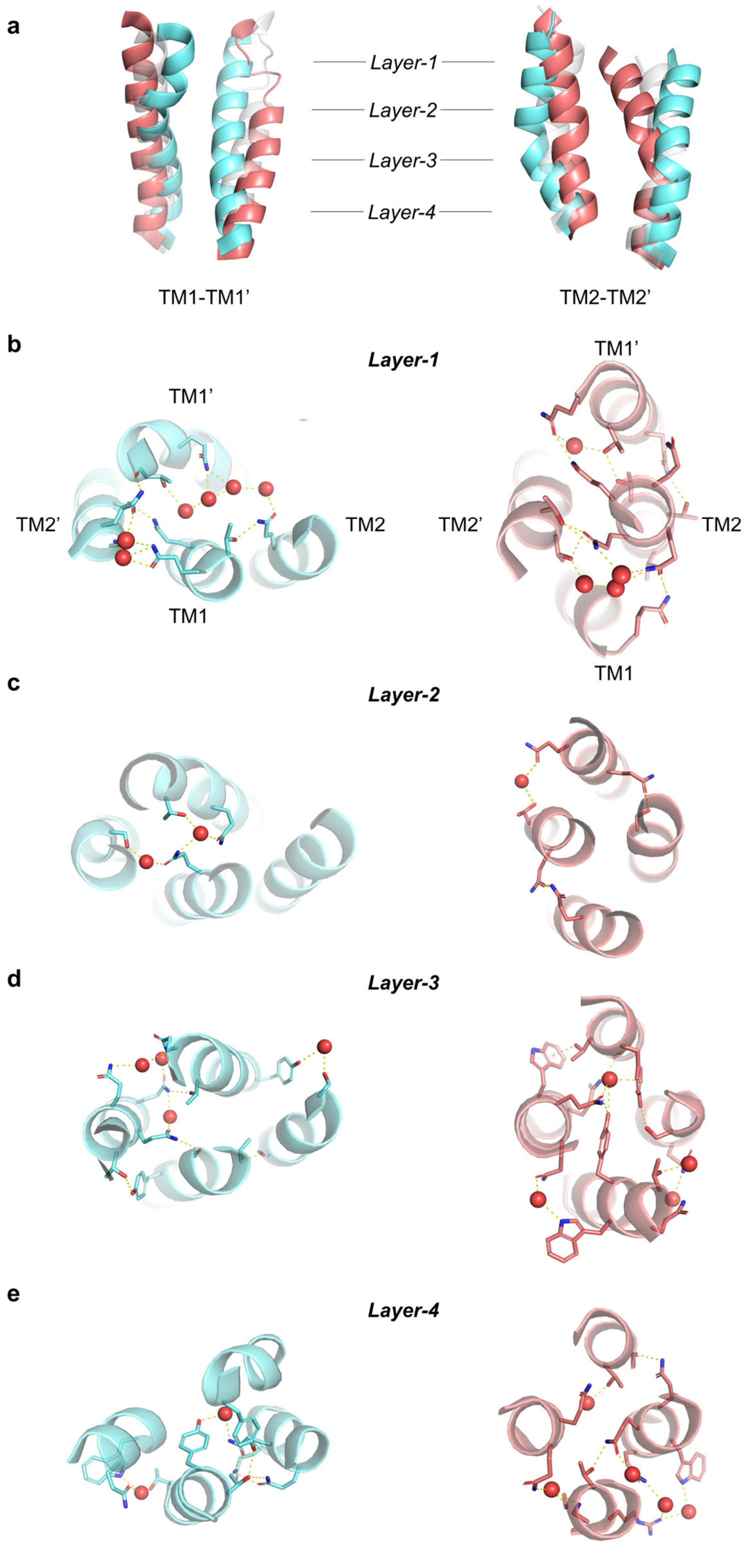

Supplementary Figure 7 | Contrast between the conformations and interhelical H-bond networks of State-1 (cyan) and State-2 (red). a, The difference of interhelical distance of TM1 (left) and TM2 helices (right). State-T was shown in transparent grey. The interaction layers were assigned, corresponding to **b-e**. **b-e**, The landscape of the interhelical H-bond network inside the transmembrane helical bundle in State-1 and State-2, shown by four layers.

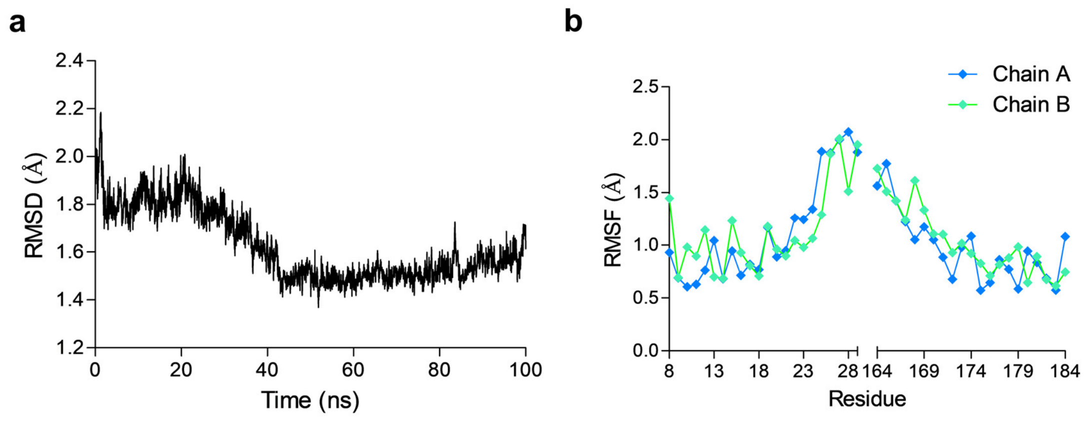

Supplementary Figure 8 | RMSD and RMSF curves in MD simulations of CpxA. **a**, Backbone RMSD of the transmembrane domain, versus medoid frame. **b**, RMSF of the transmembrane domain, versus medoid frame. Source data are provided as a Source Data file.

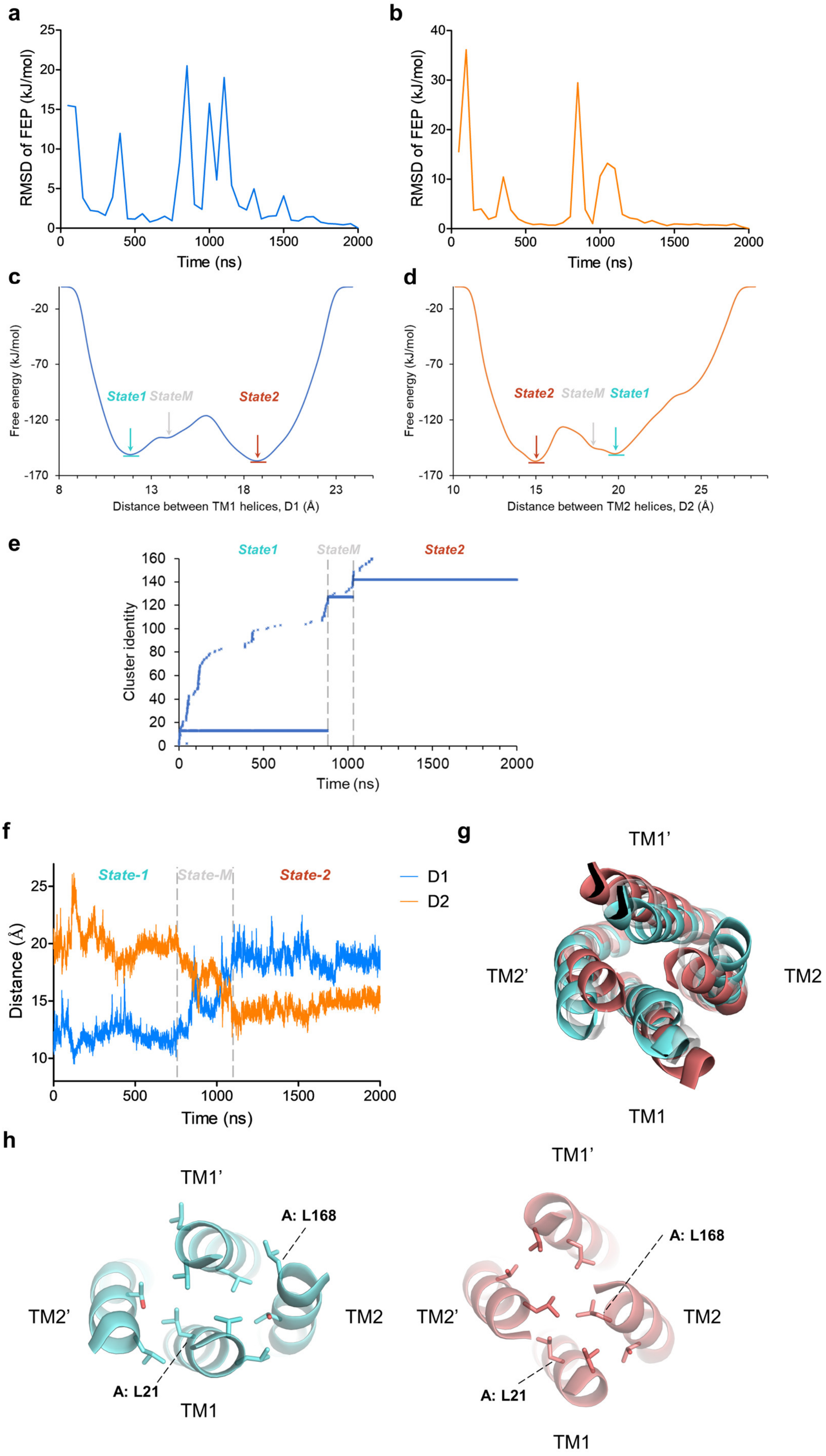

a
RMSD of FEP (kJ/mol)
Time (ns)
b
RMSD of FEP (kJ/mol)
Time (ns)
c
Free energy (kJ/mol)
State1
StateM
State2
Distance between TM1 helices, D1 (Å)
d
Free energy (kJ/mol)
State2
StateM
State1
Distance between TM2 helices, D2 (Å)
e
State1
StateM
State2
Cluster identity
Time (ns)
f
State-1
State-M
State-2
D1
D2
Distance (Å)
Time (ns)
g
TM1'
TM2'
TM2
TM1
h
TM1'
A: L168
TM2'
TM2
A: L21
TM1
TM1'
A: L168
TM2'
TM2
A: L21
TM1

Supplementary Figure 9 | PBMetaD simulation of CpxA. **a-b**, Convergence of the PBMetaD simulation was shown by RMSD of the FEP of D1 (**a**) and D2 (**b**). The FEP was aligned every 50 ns. **c-d**, Free energy profile (FEP) of D1 (**d**) and D2 (**e**). The free energy minima were indicated by arrows and classified into three states. **e**, The clustering of the conformations across the simulation time identified three major clusters, corresponding to the three states shown in CV-time curves. **f**, The curves of the CVs with simulation time. The patterns of the CV variation were classified into three states, separated by grey dashed lines. **g**, The conformational transition of the different states of the transmembrane domain. Top view of the conformations of State-1 (cyan), State-M (transparent grey) and State-2 (red), as viewed from the periplasm looking into the cytoplasm. "TM1" indicates TM1 helix in Chain A and "TM1' " indicates TM1 helix in Chain B. **h**, Snapshots of the top layer of the interaction network of State-1 (left) and State-2 (right). The display style is the same as Fig. 6d. The structural snapshots of **g** and **h** were from the medoid frames of the clusters, corresponding to **e**. Source data are provided as a Source Data file.

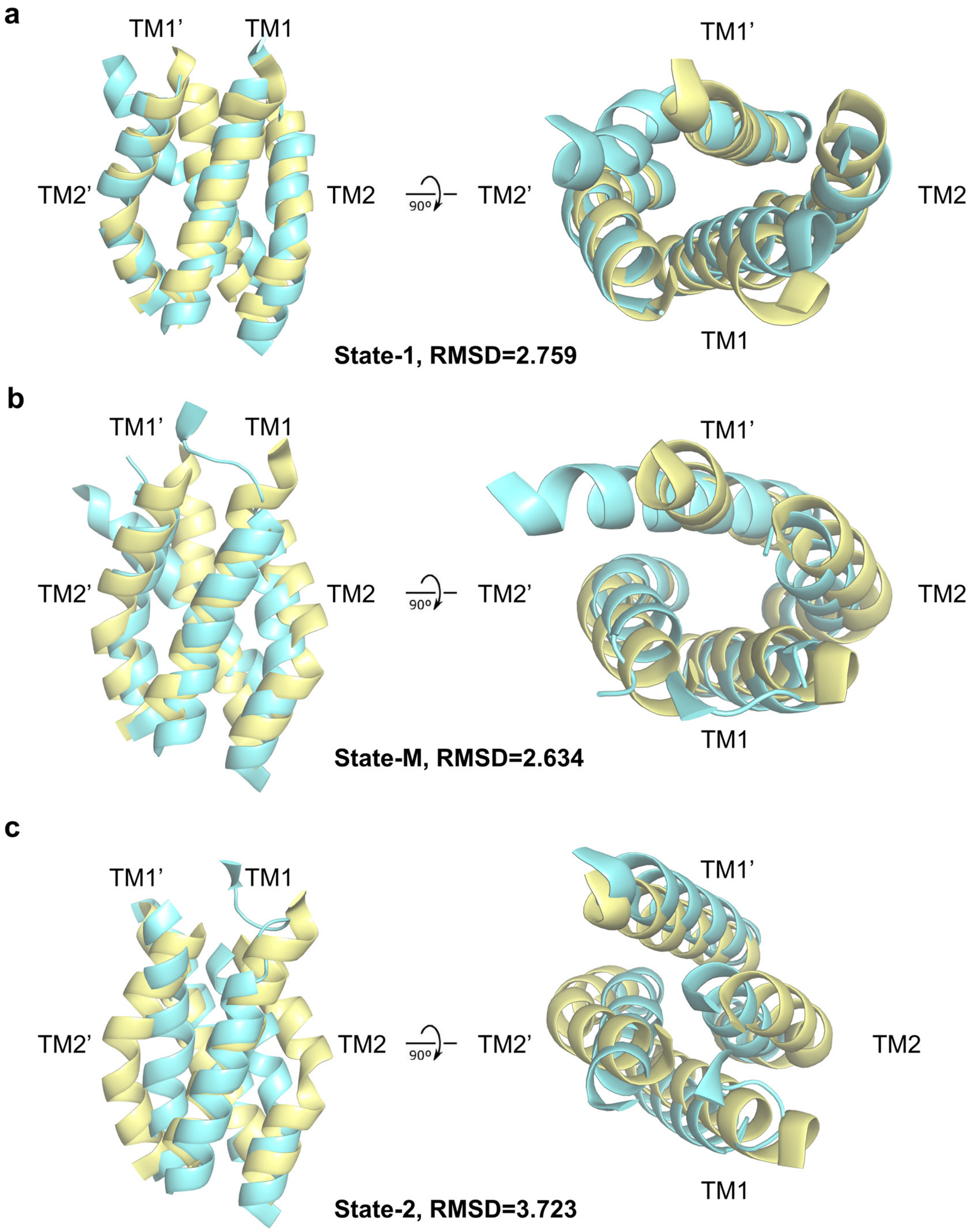

Supplementary Figure 10 | Conformational comparisons of the transmembrane domains of CpxA and CpxA^{QTY} in the corresponding stable states revealed by PBmetaD simulations. a, State-1; **b,** State-M; **c,** State-2. CpxA is in yellow and CpxA^{QTY} in cyan. Side view is shown on the left and top view right. Superpositions of the α-C atoms were conducted in PyMOL. The structural snapshots were from the simulation medoid frames. Source data are provided as a Source Data file.

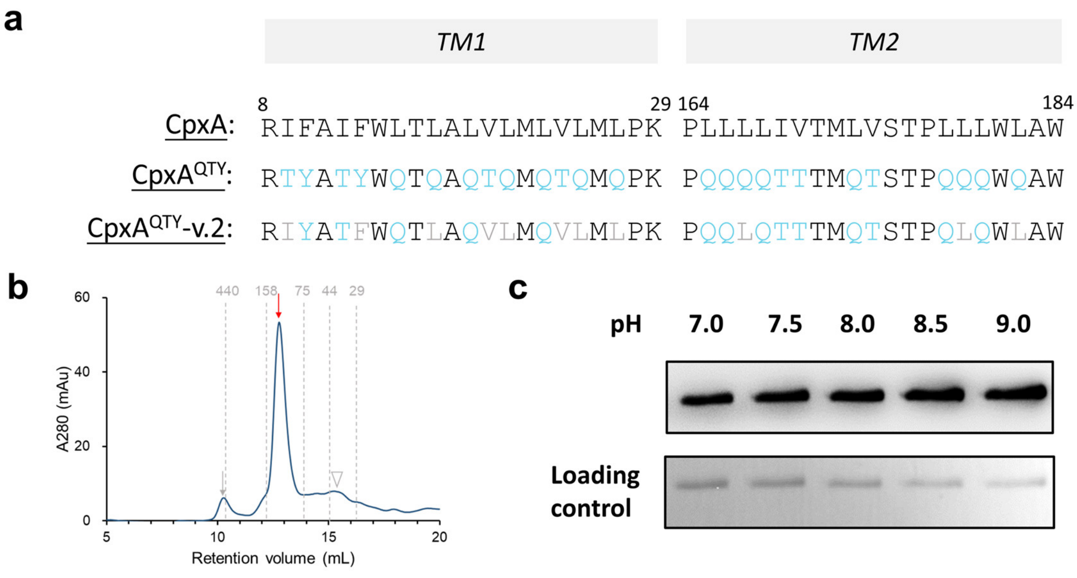

Supplementary Figure 11 | Design and characterizations of CpxA^{QTY}-v.2. a, The designed transmembrane sequence of CpxA^{QTY}-v.2, aligned with CpxA and CpxA^{QTY}. The residues applied QTY code to are in cyan and the residues of which QTY design was removed are in grey. **b**, SEC result of CpxA^{QTY}-v.2. Grey dashed lines denote the retention volumes of the standard proteins, labelled with the corresponding molecular weight (kDa). Red arrows denote the dimers, grey arrows denote aggregates and triangles denote protein impurities. The identities of the peak fractions were identified by SDS-PAGE. **c**, CpxA^{QTY}-v.2 lost the signaling activity. The same conditions as Fig. 4a. Source data are provided as a Source Data file.

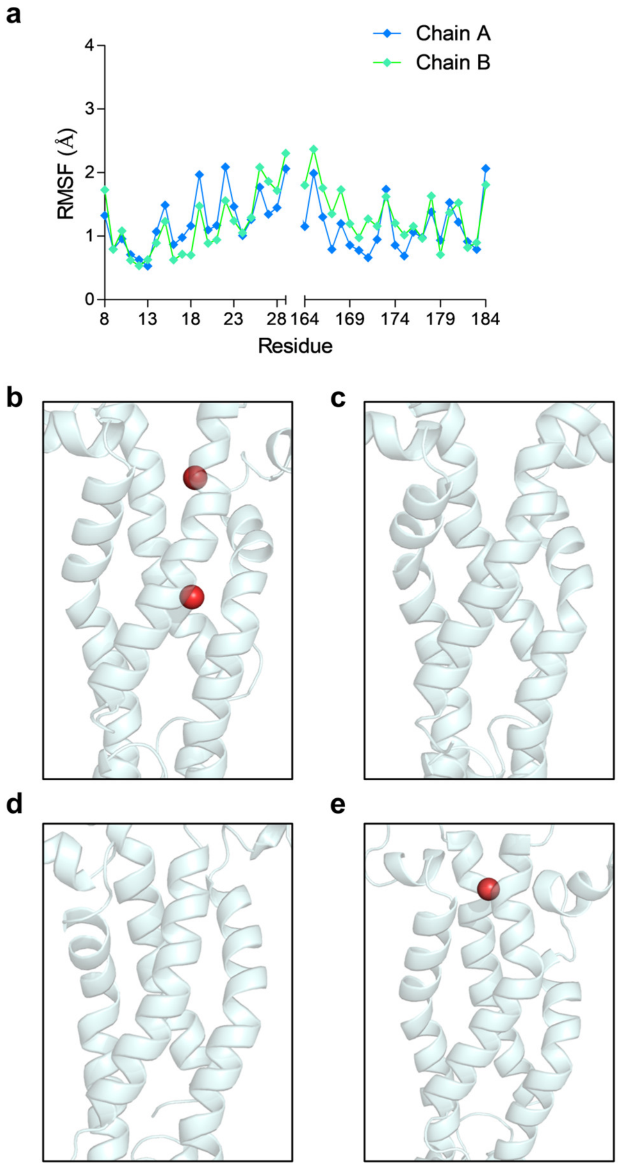

Supplementary Figure 12 | MD simulation of CpxA^{QTY}-v.2. a, RMSF of the transmembrane domain, versus medoid frame. **b-e**, Profiles of water molecule distribution inside the transmembrane domain in MD simulation of CpxA^{QTY}-v.2. Snapshots of 20 ns, 40 ns, 60 ns and 80 ns, respectively. Source data are provided as a Source Data file.

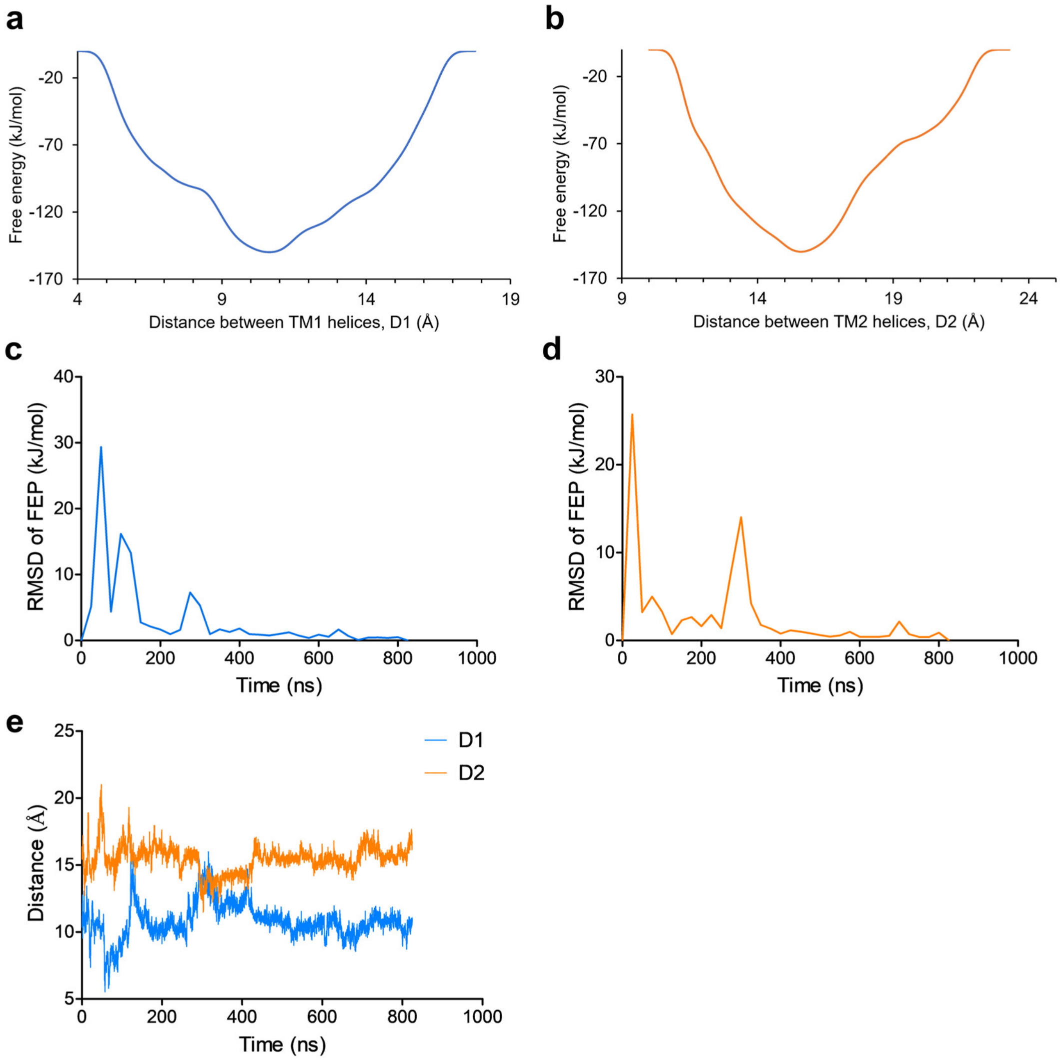

Supplementary Figure 13 | PBMetaD simulation of CpxA^{QTY}-v.2. **a-b**, Free energy profile (FEP) of D1 (**a**) and D2 (**b**). **c-d**, Convergence of the PBMetaD simulation was shown by RMSD of the FEP of D1 (**c**) and D2 (**d**). The FEP was aligned every 25 ns. **e**, The curves of the CVs with simulation time. Source data are provided as a Source Data file.

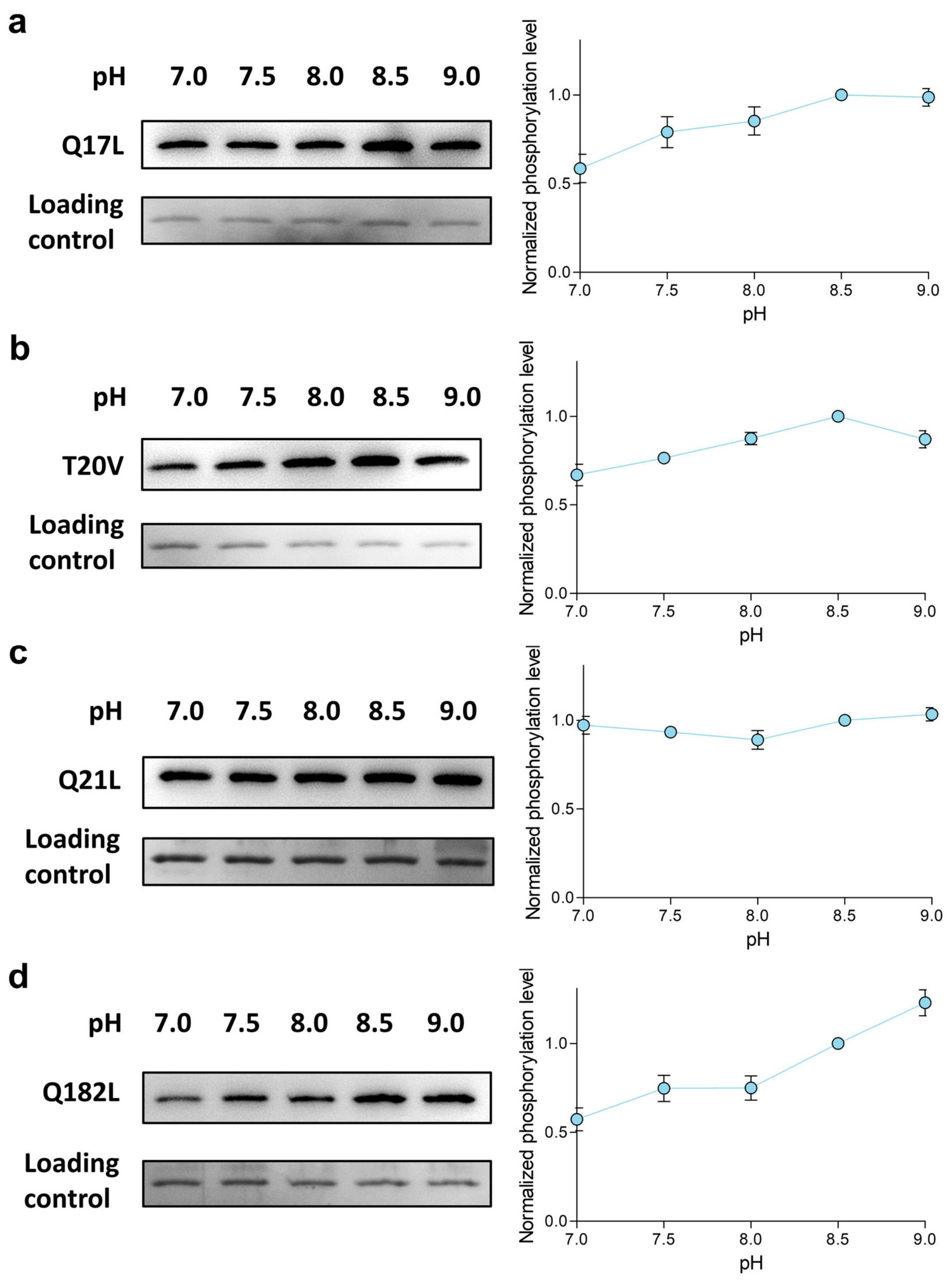

Supplementary Figure 14 | pH-sensing activities of transmembrane domain mutants of CpxA^{QTY}. a-d, Results of CpxA^{QTY}-Q17L, T20V, Q21L, and Q182L, respectively. The mutants were phosphorylated with 1 mM ATP at RT for 2 min, with indicated pH. The phosphorylation level at pH 8.5 was set as 100% for normalization. For all the three experiments, representative anti-pHis western blot results were shown (left). The quantified phosphorylation level of the three independent experiments was shown (right). Data are shown as mean ± SEM. Source data are provided as a Source Data file.

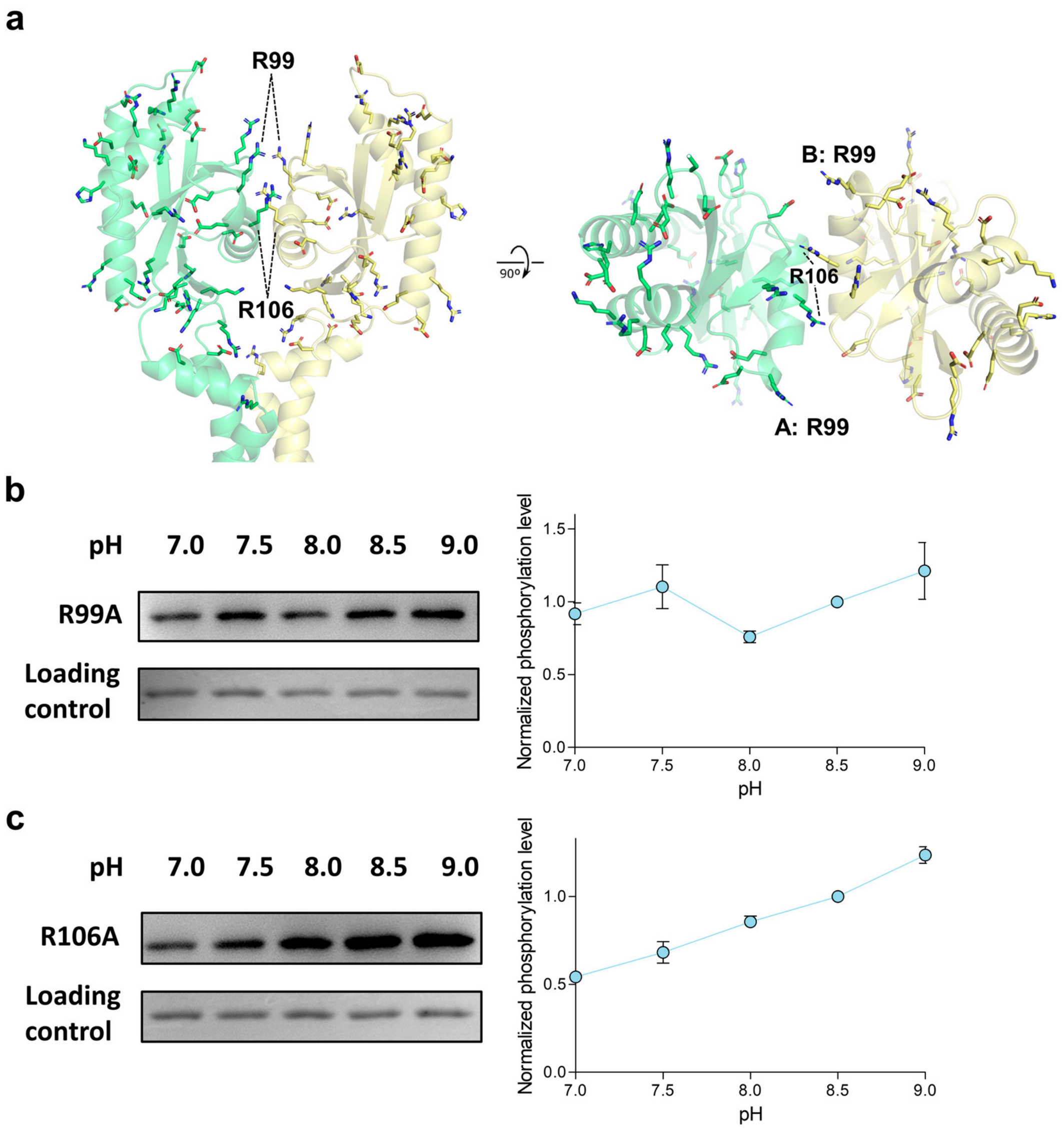

Supplementary Figure 15 | pH-sensing activities of sensor domain mutants of CpxA^{QTY}. a, Charged residues in the sensor domain. All charged residues are shown as sticks. Chain A is shown in green and Chain B in yellow. **b-c**, Results of CpxA^{QTY}-R99A and R106A, respectively. The mutants were phosphorylated with 1 mM ATP at RT for 2 min, with indicated pH. The phosphorylation level at pH 8.5 was set as 100% for normalization. For all the three experiments, representative anti-pHis western blot results were shown (left). The quantified phosphorylation level of the three independent experiments was shown (right). Data are shown as mean ± SEM. Source data are provided as a Source Data file.

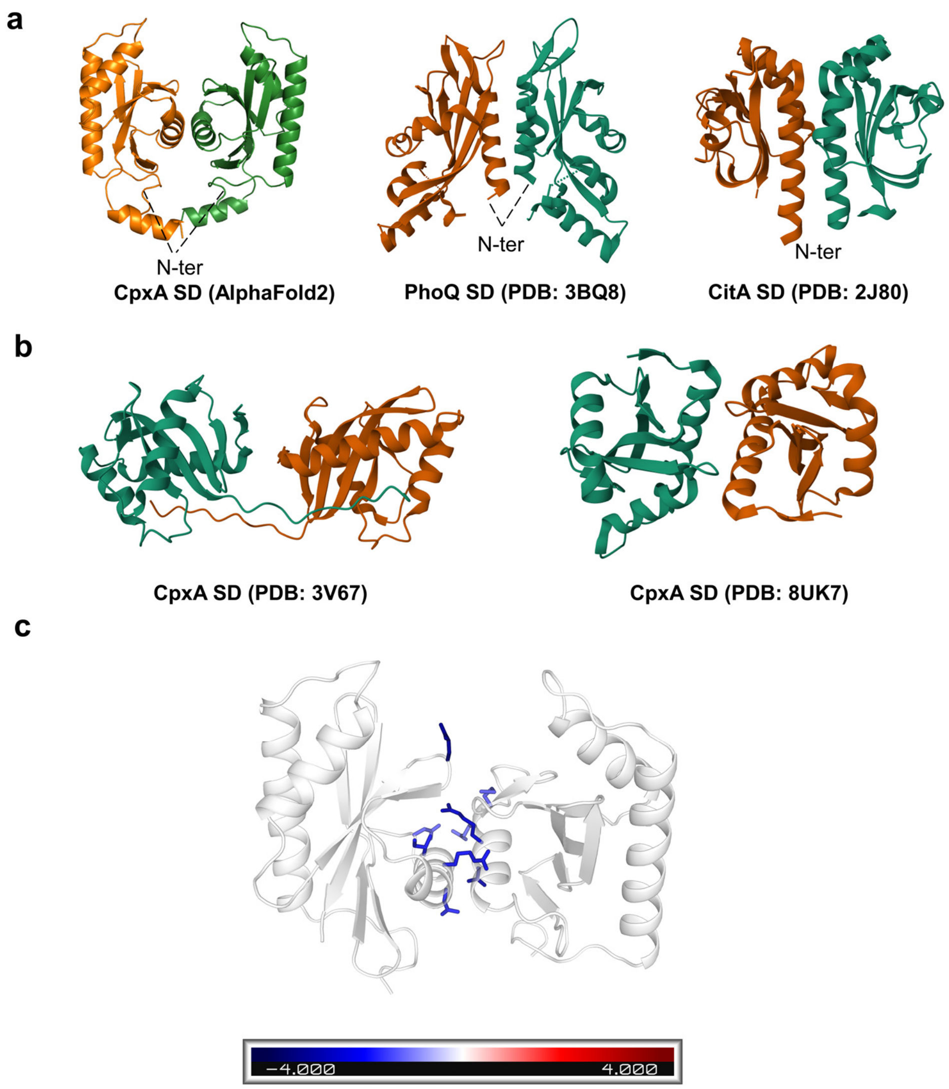

Supplementary Figure 16 | CpxA SD in the AlphaFold2 model shows a distinct dimerization mode. a, Comparison of CpxA SD dimer in the AlphaFold2 model with typical SD dimers. **b**, The crystal structures of CpxA SD. **c**, Dimer interface of CpxA SD revealed by MMPBSA binding free energy calculation. The residues that contribute (per-residue contribution > 1 or < -1 kcal/mol) to interface formation are shown as sticks, and in blue, with the color intensity determined by their contribution to binding free energy (as the bar shown below).

Supplementary Table 1. Comparison of functional performance between $CpxA^{QTY}$ in water and CpxA in lipid environment

Activity	CpxA (Ref.)	$CpxA^{QTY}$
Autokinase	Autophosphorylation was almost saturated when t=10 min (in proteoliposomes[1], detergent[2,3] and nanodiscs[4])	(Lower) Autophosphorylation was unsaturated when t=10 min, with a significantly reduced rate
Phosphotransferase	Phosphorylated CpxA became barely detectable, with CpxR still acquiring phosphate groups when t=15 min (in proteoliposomes[1], and nanodiscs[4])	(Higher) Phosphorylated $CpxA^{QTY}$ became almost undetectable when t=0.5 min, with saturation of CpxR phosphorylation reached when t=1 min
Phosphatase	Phosphorylated CpxR became barely detectable when t=30 min (in proteoliposomes[1] and detergent[3])	(Lower) Phosphorylated CpxR could still be detected when t=120 min
pH sensing	Compared to pH 7.0, the phosphorylation rate increased by ~ 2.5-fold at pH 7.5 and by ~ 4-fold at pH 8.0 (in proteoliposomes[1])	(Slightly higher) Compared to pH 7.0, the phosphorylation rate increased by ~ 2.7-fold at pH 7.5 and by ~ 7.5-fold at pH 8.0
CpxP sensing	Phosphorylation rate was inhibited by ~50% when with equimolar CpxP (in proteoliposomes[1])	(Lower) Phosphorylation rate was inhibited by ~50% when with 10-fold CpxP
K^+ sensing	Minor response to 0.5 mM and major response to 500 mM (in proteoliposomes[1])	(Lower) Approximate response range was 10 to 100 mM

Note: For CpxA, the types of the lipid environment used in the references were indicated at the end of description; for $CpxA^{QTY}$, the apparent relative level of its activities compared to CpxA was indicated at the beginning.

Supplementary Table 2. Primer sequences

Primer	Sequence (5'-3')
CpxP-F	AACTTTAAGAAGGAGATATACCATGGCTGAAGTCGGTTCAGGC
CpxP-R	AGTGGTGGTGGTGGTGGTGCTCGAGCTGGGAACGTGAGTTGCTA
CpxR-F	AACTTTAAGAAGGAGATATACCATGAATAAAATCCTGTTAGTTGA TGATGACC
CpxR-R	AGTGGTGGTGGTGGTGGTGCTCGAGTGAAGCAGAAACCATCAG ATAGC
CpxAc-F	TGCCGCGCGGCAGCCATATGAAACCGGCCCGCAAA
CpxAc-R	TGGTGGTGGTGGTGCTCGAGTTAGCTGCGTTTGTACAGCGG
CpxAQ-Strep-F	TGGTCTCACCCGCAGTTCGAAAAAATCGGCAGCCTGACCGC
CpxAQ-Strep-R	TTTTTCGAACTGCGGGTGAGACCACATGTATATCTCCTTCTTAAA GTTAAACAA
CpxAQ-Q17L-F	TGGCAGACCCTGGCCCAAACCCAGATGCAGAC
CpxAQ-Q17L-R	GGTTTGGGCCAGGGTCTGCCAGTAGGTGGCGT
CpxAQ-T20V-F	CAGGCCCAAGTTCAGATGCAGACCCAAATGCA
CpxAQ-T20V-R	CTGCATCTGAACTTGGGCCTGGGTCTGCCAGT
CpxAQ-Q21L-F	GCCCAAACCCTGATGCAGACCCAAATGCAGCC
CpxAQ-Q21L-R	GGTCTGCATCAGGGTTTGGGCCTGGGTCTGCC
CpxAQ-Q182L-F	CAACAGTGGCTGGCCTGGAGCCTGGCCAAACC
CpxAQ-Q182L-R	GCTCCAGGCCAGCCACTGTTGTTGCGGGGTGCT
R99A-F	GGCGCCGAAGCCAGCGAAATGCAGATCATCCGCAA
R99A-R	CATTTCGCTGGCTTCGGCGCCGATAACGCGGC
R106A-F	CAGATCATCGCCAATTTCATCGGCCAGGCCGATA
R106A-R	GATGAAATTGGCGATGATCTGCATTTCGCTGC

Supplementary Discussion

Typically, the SD dimerizes via the first helix (starting from the N-terminus), whereas in this model it dimerizes via the third helix (Supplementary Fig. 16a). There are two released crystal structures of CpxA SD: one is PDB: 3V67, from *Vibrio parahaemolyticus*[5]; the other is recently-released PDB: 8UK7, from *E. coli* (Supplementary Fig. 16b). We noticed that two significant differences between CpxA SD and that of other typical SHKs: 1) its N-terminus forms a 10-residue loop before the first helix rather than a continuous helix connected with the transmembrane helix; 2) the crystal structures of CpxA SD only revealed the monomeric structure (in fact they are dimeric, but they are crystallographic dimers), rather than dimeric structure like most resolved SD crystal structures. The failure of both the two CpxA SD crystals in forming a bona fide dimer suggests that the dimerization might be not as strong as that of others. We inferred that the dimerization of CpxA SD might be distinct from the typical SD dimerization.

To validate the reliability of the untypical sensor domain (SD) dimerization in the AlphaFold2 model, we conducted binding free energy (BFE) calculation using the Molecular Mechanics Poisson–Boltzmann Surface Area (MMPBSA) algorithm. The results showed a decent binding strength (BFE = −36.25 ± 0.09 kcal/mol). We observed eight interface residues that significantly contribute to the binding (per-residue contribution < −1 kcal/mol), and no interface residues that show negative contributions (Supplementary Fig. 16c). These results suggest that the dimerization mode in the AlphaFold2 model is reliable.

Supplementary References

1. Fleischer, R., Heermann, R., Jung, K. & Hunke, S. Purification, reconstitution, and characterization of the CpxRAP envelope stress system of Escherichia coli. *J Biol Chem* **282**, 8583-93 (2007).
2. Mechaly, A.E., Sassoon, N., Betton, J.M. & Alzari, P.M. Segmental helical motions and dynamical asymmetry modulate histidine kinase autophosphorylation. *PLoS Biol* **12**, e1001776 (2014).
3. Miot, M. & Betton, J.M. Reconstitution of the Cpx signaling system from cell-free synthesized proteins. *N Biotechnol* **28**, 277-81 (2011).
4. Hörnschemeyer, P., Liss, V., Heermann, R., Jung, K. & Hunke, S. Interaction Analysis of a Two-Component System Using Nanodiscs. *PLoS One* **11**, e0149187 (2016).
5. Kwon, E. et al. The crystal structure of the periplasmic domain of Vibrio parahaemolyticus CpxA. *Protein Sci* **21**, 1334-43 (2012).
