# Supplementary material for: Design of a water-soluble transmembrane receptor kinase with intact molecular function by QTY code
Source: Nat Commun. 2024 Jun 10;15:4293. doi: 10.1038/s41467-024-48513-9 (PMC11164701; doi:10.1038/s41467-024-48513-9)
Supplement: Supplementary file 3 — Reporting Summary [file 41467_2024_48513_MOESM3_ESM.pdf]

nature portfolio

Corresponding author(s): Fei Tao, Shuguang Zhang, Ping Xu

Last updated by author(s): Apr 29, 2024

Reporting Summary

Nature Portfolio wishes to improve the reproducibility of the work that we publish. This form provides structure for consistency and transparency in reporting. For further information on Nature Portfolio policies, see our Editorial Policies and the Editorial Policy Checklist.

Statistics

For all statistical analyses, confirm that the following items are present in the figure legend, table legend, main text, or Methods section.

n/a	Confirmed	
[ ]	[x]	The exact sample size (n) for each experimental group/condition, given as a discrete number and unit of measurement
[ ]	[x]	A statement on whether measurements were taken from distinct samples or whether the same sample was measured repeatedly
[x]	[ ]	The statistical test(s) used AND whether they are one- or two-sided<br>*Only common tests should be described solely by name; describe more complex techniques in the Methods section.*
[x]	[ ]	A description of all covariates tested
[x]	[ ]	A description of any assumptions or corrections, such as tests of normality and adjustment for multiple comparisons
[ ]	[x]	A full description of the statistical parameters including central tendency (e.g. means) or other basic estimates (e.g. regression coefficient) AND variation (e.g. standard deviation) or associated estimates of uncertainty (e.g. confidence intervals)
[x]	[ ]	For null hypothesis testing, the test statistic (e.g. F, t, r) with confidence intervals, effect sizes, degrees of freedom and P value noted<br>*Give P values as exact values whenever suitable.*
[x]	[ ]	For Bayesian analysis, information on the choice of priors and Markov chain Monte Carlo settings
[x]	[ ]	For hierarchical and complex designs, identification of the appropriate level for tests and full reporting of outcomes
[x]	[ ]	Estimates of effect sizes (e.g. Cohen's d, Pearson's r), indicating how they were calculated

Our web collection on statistics for biologists contains articles on many of the points above.

Software and code

Policy information about availability of computer code

Data collection	ColabFold (1.0.0) was used for structure model generation; GROMACS 2021.3 was used for classical MD simulations; GROMACS 2022.3 and PLUMED 2.8.1 were used for PBMetaD simulations; PyMOL (2.5) and Chimera (1.16) were used for protein structure processing, analysis and figure-making.
Data analysis	SEDFIT (15.10b) and GUSSI (1.0.8) were used for analytical ultracentrifugation data analysis; PR.ThermoControl was used for nanoDSF data analysis; The LC/MS software Data Analysis (4.0), MASCOT (2.3) and Protein Discovery (2.5) were used for mass spectrometry data analysis; GraphPad Prism (5.01) was used for data visualization and figure-making. JASCO Spectra Manager (2.0) was used for circular dichroism data analysis.

For manuscripts utilizing custom algorithms or software that are central to the research but not yet described in published literature, software must be made available to editors and reviewers. We strongly encourage code deposition in a community repository (e.g. GitHub). See the Nature Portfolio guidelines for submitting code & software for further information.

Data

Policy information about availability of data

All manuscripts must include a data availability statement. This statement should provide the following information, where applicable:

- Accession codes, unique identifiers, or web links for publicly available datasets
- A description of any restrictions on data availability
- For clinical datasets or third party data, please ensure that the statement adheres to our policy

The simulation data generated in this study have been deposited in the Zenodo database under accession code 10516203. All other data generated in this study are provided in the Supplementary Information/Source Data file. The CpxA sequence used in this study are available in the Uniprot database under accession P0AE82. Source data are provided with this paper.

Research involving human participants, their data, or biological material

Policy information about studies with human participants or human data. See also policy information about sex, gender (identity/presentation), and sexual orientation and race, ethnicity and racism.

Reporting on sex and gender	Not involving human participants
Reporting on race, ethnicity, or other socially relevant groupings	Not involving human participants
Population characteristics	Not involving human participants
Recruitment	Not involving human participants
Ethics oversight	Not involving human participants

Note that full information on the approval of the study protocol must also be provided in the manuscript.

Field-specific reporting

Please select the one below that is the best fit for your research. If you are not sure, read the appropriate sections before making your selection.

☒ Life sciences ☐ Behavioural & social sciences ☐ Ecological, evolutionary & environmental sciences

For a reference copy of the document with all sections, see nature.com/documents/nr-reporting-summary-flat.pdf

Life sciences study design

All studies must disclose on these points even when the disclosure is negative.

Sample size	All samples were tested in triplicate or more (standard for protein science).
Data exclusions	No data excluded.
Replication	All attempts at replication were successful.
Randomization	This is not relevant for studying protein samples in vitro.
Blinding	This is not relevant for studying protein samples in vitro.

Reporting for specific materials, systems and methods

We require information from authors about some types of materials, experimental systems and methods used in many studies. Here, indicate whether each material, system or method listed is relevant to your study. If you are not sure if a list item applies to your research, read the appropriate section before selecting a response.

Materials & experimental systems

n/a	Involved in the study	
☐	☒	Antibodies
☒	☐	Eukaryotic cell lines
☒	☐	Palaeontology and archaeology
☒	☐	Animals and other organisms
☒	☐	Clinical data
☒	☐	Dual use research of concern
☒	☐	Plants

Methods

n/a	Involved in the study	
☒	☐	ChIP-seq
☒	☐	Flow cytometry
☒	☐	MRI-based neuroimaging

Antibodies

Antibodies used

Anti-N3-Phosphohistidine Antibody, clone SC39-6, ZRB1351, Sigma-Aldrich; goat anti-rabbit IgG-HRP conjugate (Absin, abs20040)

Validation

Biological source: rabbit; Species reactivity: human; Application:Anti-N3-Phosphohistidine (3-pHis), clone SC39-6 ZooMAb, Cat. No. ZRB1351, is a recombinant Rabbit Monoclonal Antibody that targets N3-Phosphohistidine and has been tested for use in Western Blotting; Enhanced validation: recombinant expression.
